# *HLA-G*, *LILRB1* and *LILRB2* Variants in Zika Virus Transmission from Mother to Child in a Population from South and Southeast of Brazil

**DOI:** 10.3390/cimb44070191

**Published:** 2022-06-27

**Authors:** Amarilis Giaretta de Moraes, Christiane Maria Ayo, Laise Nayana Sala Elpídio, Victor Hugo de Souza, Aléia Harumi Uchibaba Yamanaka, Maurício Lacerda Nogueira, Saulo Duarte Passos, Cinara Cássia Brandão, Luiz Carlos de Mattos, Greicy Cezar do Amaral, Quirino Alves de Lima Neto, Jeane Eliete Laguila Visentainer

**Affiliations:** 1 Post Graduation Program in Biosciences and Physiopathology, Department of Clinical Analysis and Biomedicine, State University of Maringá, Maringá 87020-270, PR, Brazil; laise_nayana@hotmail.com (L.N.S.E.); victoruem@gmail.com (V.H.d.S.); aleiayamanaka@hotmail.com (A.H.U.Y.); qalneto@uem.br (Q.A.d.L.N.); 2 Laboratory of Immunogenetics, Department of Basic Health Sciences, State University of Maringá, Maringá 87020-270, PR, Brazil; 3 Laboratory of Immunogenetics, Department of Molecular Biology, Faculty of Medicine of São José do Rio Preto (FAMERP), São José do Rio Preto 15090-000, SP, Brazil; chris.ayo@hotmail.com (C.M.A.); cinara.brandao@edu.famerp.br (C.C.B.); luiz.demattos@edu.famerp.br (L.C.d.M.); 4 Virology Research Laboratory, Department of Infectious and Parasitic Diseases, Medical School of São José do Rio Preto (FAMERP), São José do Rio Preto 15090-000, SP, Brazil; mauricio.nogueira@edu.famerp.br; 5 Department of Pediatrics, Faculty of Medicine of Jundiai (FMJ), Jundiaí 13202-550, SP, Brazil; sauloduarte@uol.com.br; 6 15th Regional of Health of the State of Paraná, Maringá 87030-090, PR, Brazil; greicyc@sesa.pr.gov.br

**Keywords:** ZIKV, vertical transmission, SNPs, flavivirus, HLA, LILRB

## Abstract

During the 2015–2016 epidemic, Brazil was the country with the highest rate of Zika virus (ZIKV) infection in the Americas. Twenty-nine percent of pregnant women positive for ZIKV exhibited ultrasound scans with fetus anomalies. Human leukocyte antigen-G (HLA-G) exerts immunoregulatory effects by binding to inhibitory receptors, namely LILRB1 and LILRB2, thus preventing mother–fetus rejection and vertical pathogen transmission. The binding of HLA-G to one of its receptors modulates both innate and adaptive immunity. However, in a viral infection, these molecules may behave as pathogenic mediators shifting the pregnancy environment from an anti-inflammatory profile to a pro-inflammatory phenotype. Genetic mutations might be associated with the change in phenotype. This study aimed to explore the possible role of polymorphic sites in *HLA-G*, *LILRB1* and *LILRB2* in mother–fetus ZIKV transmission. Polymorphisms were detected by direct sequencing. Differences in allele and/or genotype frequencies for each SNP analyzed among ZIKV non-transmitting and transmitting mother–child pairs, among ZIKV-transmitting and non-transmitting mothers and between ZIKV-infected and non-infected children were compared by Mid-P exact test or Yates’ correction. Significant susceptibility of ZIKV vertical transmission is suggested in ZIKV-transmitting and non-transmitting mothers and ZIKV-infected and non-infected children for *LILRB1_rs1061684 T*/*T* (*p* = 0.03, *Pc* = 0.06, OR = 12.4; *p* = 0.008, *Pc* = 0.016, OR = 16.4) and *LILRB1_rs16985478 A*/*A* (*p* = 0.01, *Pc* = 0.02, OR = 19.2; *p* = 0.008, *Pc* = 0.016, OR = 16.4). *HLA-G_rs1710* (*p* = 0.04, *Pc* = 0.52, OR = 4.30) was also a susceptibility factor. *LILRB2_rs386056 G*/*A* (*p* = 0.02, *Pc* = 0.08, OR = 0.07), *LILRB2_rs7247451 G*/*G* (*p* = 0.01, *Pc* = 0.04, OR = 0.04) and *HLAG_rs9380142 T*/*T* (*p* = 0.04, *Pc* = 0.52, OR = 0.14) were suggested as protective factors against vertical transmission. The current study suggests that polymorphic sites in the *LILRB1* and *HLA-G* genes might be associated with mother-to-child ZIKV transmission while *LILRB2* might be associated with protection against ZIKV transmission in the womb in a population from the south and southeast of Brazil.

## 1. Introduction

ZIKV is a flavivirus member of the *Flaviviridae* family and is closely related to dengue virus (DENV), West Nile virus (WNV) and others [1,2]. The outbreak in the Americas in early 2015 emerged as a major human threat because of its correlation with the increased number of newborns with congenital Zika syndrome (CZS) [3]. Brazil was the country with the highest rate of ZIKV infections in the continent [3,4]. It was reported that 29% of pregnant women who tested positive for ZIKV exhibited ultrasound scans with fetus abnormalities [5]. The contemporaneous ZIKV strains belong to Asian lineage and are primarily responsible for human outbreaks [6]. However, experimental evidence has demonstrated that the African strains exhibit higher epidemic potential, with increased transmissibility and fetus lethality compared to the Asian strains [6]. Most worryingly, both ZIKV lineages can be vertically transmitted [6].

The ZIKV genome was detected in amniotic fluid and placental and fetal brain tissues, and vertical transmission was proposed as a possible route [7,8,9,10,11]. Although there is a strong correlation between maternal ZIKV transmission to the unborn, the molecular mechanisms by which ZIKV crosses the placenta barrier and infects the fetus are yet unclear. This drives us to think that factors associated with the maternal–fetal interface can modulate this relationship, be tissue-specific and play an important role during pregnancy and viral transmission. In this regard, a growing body of evidence indicates that the nonclassical human leukocyte antigen-G (HLA-G), which is highly expressed in trophoblast cells and viral infections, has a crucial immune modulatory role. HLA-G exerts its immunoregulatory effects by binding to specific inhibitory receptors on different types of immune cells and preventing mother–fetus rejection as well as vertical pathogen transmission [12]. Leukocyte immunoglobulin-like receptors (LILRB1 and LILRB2) present a high affinity to HLA-G ligand in the lower-intensity killer cell immunoglobulin-like receptor 2DL4 (KIR2DL4) [12,13]. The binding of HLA-G to one of its receptors can modulate both innate and adaptive immune responses [12]. However, in the presence of a viral infection, these molecules may behave as pathogenic mediators shifting the pregnancy environment from an anti-inflammatory profile to a pro-inflammatory phenotype, thus making it more susceptible to pathogens [12,13]. Genetic mutations might also be associated with the change in phenotype.

Several studies have shown the role of HLA-G variants in the risk of developing HIV infection and in vertical transmission. In a mother–child study, it was reported that discordant *HLA-G* polymorphisms in the *3′ untranslated region* (*3′UTR*), such as *rs1710* and the *14bp insertion*/*deletion* (*rs371194629*), were associated with a minor risk of HIV transmission from mother to child [14]. Another study identified that carriers of the *14bp insertion* allele are linked to lower HLA-G molecule expression in the placenta [14,15,16]. A Brazilian study investigating the role of *HLA-G* polymorphisms in HIV vertical transmission found a protective effect of the *14bp Deletion*/*Deletion* against perinatal transmission [17]. Additionally, *HLA-G* haplotypes have been reported as a protective factor against mother–child HIV transmission [18].

Considering that pregnancy is a critical stage of human formation—and due to ethical policies—experiments and testing for ZIKV can become very difficult or even impossible; furthermore, murine models are not useful to study HLA-G due to its uncertainty of existence. This highlights the necessity of using alternative ways to support the diagnostic methods already used. Therefore, this study aimed to examine the possible role of polymorphic sites in the alpha 1–3 domains and the *3′ UTR* of *HLA-G* and variants in *LILRB1* and *LILRB2* in mother–fetus ZIKV transmission.

## 2. Materials and Methods

A total of 20 mother–child pairs were recruited from Jundiaí, São Paulo and the northern region of the state of Paraná, Brazil, which included 14 ZIKV-infected mother–child pairs (one mother had twins) and 6 ZIKV-infected mother–uninfected child pairs. The mean age in the ZIKV-infected mother–child group was 29.92 ± 9.23 and that in the ZIKV-infected mother–uninfected child group was 28.67 ± 5.47. The expectant mothers from Jundiaí sought medical care at the University Hospital of the Faculty of Medicine of Jundiaí. Pregnant subjects from the Paraná region were attended to in the closest medical care unit of the public health system. The expectant mothers sought medical care with ZIKV-related symptoms during the ZIKV outbreak in 2015/2016.

According to the World Health Organization (WHO), a person is classified as a suspected case of ZIKV infection when presenting rash and/or fever and one of the following symptoms: arthralgia, arthritis, conjunctivitis or pruritus. The confirmation of a case of ZIKV infection was based on molecular or serological tests [19]. Laboratory tests were performed at the University Hospital of the Faculty of Medicine of Jundiaí and by the Central Laboratory of the State of Paraná. Both establishments were reference laboratories for testing ZIKV by the quantitative molecular technique of reverse transcription polymerase chain reaction (RT-qPCR). Children were tested at the time of delivery from the following biological specimens: tear secretion, serum, umbilical cord, placenta or saliva (when available).

All participants in this study were informed and signed the written consent form. Adults responsible for their children also signed the written consent form. This study was approved by the Human Research and Ethics Committee of the Faculty of Medicine of Jundiaí (CAAE 53248616.2.0000.5412), of the Medical School of São José do Rio Preto (CAAE 55805516.2.0000.5415) and of the State University of Maringá (CAAE 64338116.1.000.0104).

### 2.1. DNA Extraction and PCR Amplification

Genomic DNA was isolated from whole blood or a buffy coat layer using the QIAamp DNA purification Mini Kit (QIAGEN, The Netherlands) according to the manufacturer’s instructions. The *HLA-G exons 2–4*, *HLA-G exon 8* (3′ UTR), *LILRB1* and *LILRB2* were PCR amplified using the primers described in Supplementary Material S1 (Table S1.1.). All PCR amplifications were performed in a final volume of 20 µL containing 1X PCR buffer (0.1 M Tris-HCl, pH 8.8; 0.5 M KCl), 0.25 mM of dNTP Mix, 1 mM (*HLA-G*) or 1.5 mM (*LILRB1* and *LILRB2*) of MgCl_2_, 0.15 µM (*HLA-G* and *LILRB1*) or 0.25 µM (*LILRB2*) of each primer, 0.8 units of Taq DNA-polymerase (*HLA-G exon 8*, *LILRB1* and *LILRB2)* or Platinum Taq DNA-polymerase (*HLA-G exons 2–4*) (Invitrogen, Carlsbad, CA, USA) and 75 ng of genomic DNA. The thermal cycling conditions for each gene fragment are shown in Supplementary Material S1 (Table S1.2). The PCR products were visualized in 2% agarose gel stained with SYBR™ Safe stain (Invitrogen, Carlsbad, CA, USA).

### 2.2. Sanger Sequencing

The amplification products were first diluted approximately 10× before directly sequencing in an ABI3500xl Genetic Analyzer (Applied Biosystems, Foster City, CA, USA) using POP-7^TM^ polymer and a 50-centimeter array. The sequencing reaction was carried out using a BigDye™ Terminator v3.1 Cycle Sequencing Kit (Applied Biosystems—Thermo Fisher Scientific, Waltham, MA, USA) following the manufacturer’s recommendation, in a final volume of 10 µL. Forward and reverse primers were used for each gene fragment until optimization of the sequencing reaction (Supplementary Material S1—Tables S1.1. and S1.3.). After optimization, only the primer (forward or reverse) with the lowest or no background and that did not overlap the sequences of the PCR primers was chosen (Supplementary Material S1—Tables S1.1. and S1.3.). Purification of the sequencing reaction was performed using the Ethanol/EDTA precipitation method following the protocol available in the BigDye™ Terminator v3.1 user guide. Purified and dried sequencing reactions were resuspended in 10 μL of Hi-Di^TM^ Formamide before subjecting the samples to capillary electrophoresis. All polymorphic sites identified were individually annotated.

### 2.3. Data Analysis

We used the following definitions of similarity/dissimilarity for *LILRB1*, *LILRB2* and *HLA-G* variants: a mother–child pair was classified as similar for a specific genetic variant when both the mother and child presented the same genotype, homozygous or heterozygous. Dissimilarity was considered when the mother–child pair presented different genotypes.

A second comparison of alleles and genotype frequencies was performed to evaluate the possible associations of *LILRB1*, *LILRB2* and *HLA-G* polymorphisms with the ZIKV-transmitting and non-transmitting mothers and between ZIKV-infected and non-infected children.

The statistical analyses were performed using SNPstats (https://www.snpstats.net, accessed on 22 February 2022) and OpenEpi version 3 (http://www.openepi.com/OE2.3/Menu/OpenEpiMenu.htm, accessed on 23 February 2022) to determine the allelic and genotypic frequencies and to calculate the differences between allelic and genotypic frequencies in each of the gene variants analyzed, respectively, in the following groups: ZIKV non-transmitting mother–child pairs and ZIKV-transmitting mother–child pairs, ZIKV-transmitting and non-transmitting mothers and ZIKV-infected and non-infected children.

Frequencies were calculated and compared by Mid-P exact test or Yates’ correction with chi-squared analysis when necessary, using two-tailed *p*-values, odds ratios (ORs) and 95% confidence intervals (Cis). The values were considered statistically significant at *p* < 0.05. The Bonferroni correction (BC) was applied to manage the multiple tests issue. Although the BC must be calculated by establishing a new cut-off point for the *p*-value, we opted to use the conventional method in which we multiply the *p*-value by the number of SNPs tested in each gene. This makes it easier to observe the correction. In addition, we also chose to analyze each gene as an independent test, as they were considered in the formulation of our hypotheses. Therefore, the corrected *p*-value (*Pc*) was considered significant when *Pc* < 0.05. An inheritance model analysis was also performed in SNPstats software. To choose the best model that fits our data, the Akaike information criterion (AIC) with the lowest value was used. Five inheritance models were evaluated: codominant, dominant, recessive, overdominant and additive. Genotype distribution was estimated and tested for the Hardy–Weinberg equilibrium (HWE) using SNPstats software.

## 3. Results

### 3.1. Association of HLA-G and LILRB1/2 Polymorphisms in Similar/Dissimilar Groups

**Figure 1:**
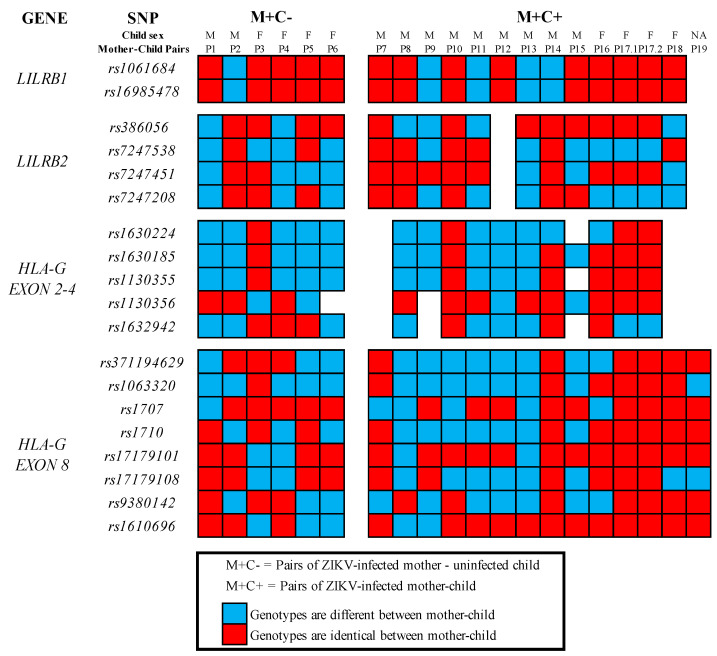
Graphical representation of similarity and dissimilarity genotypes for each gene variant analyzed among ZIKV non-transmitting mother–child and ZIKV-transmitting mother–child pairs.

A total of 19 variants in the genes *LILRB1*, *LILRB2* and *HLA-G* were analyzed to verify the association of polymorphisms with ZIKV vertical transmission. The following polymorphisms were evaluated: two SNPs in *LILRB1 exon 15*, namely *rs1061684* (−5717 *C > T*) and *rs16985478* (−5724 *G > A*); four SNPs in *exon 6* of *LILRB2*, namely *rs386056* (+703 *G > A*), *rs7247538*, *rs7247451* and *rs7247208*; five polymorphisms within *exons 2–4*, namely *rs1630224* and *rs1630185* (+36 *G > A*) in *HLA-G exon 2*, *rs1130355* (+372 *G > A*) in *HLA-G exon 3* and *rs1130356* (+706 *C > T*) and *rs1632942* (+1019 *T > C*) in *HLA-G exon 4*; and a total of eight genetic variants in *HLA-G exon 8 (3′UTR)*, namely *rs371194629* (*14 bp Ins*/*Del*), *rs1063320* (+ 3142 *C*/*G*), *rs1707* (+3003 *A*/*G*), *rs1710* (+3010 *C*/*G*), *rs17179101* (+3027 *T*/*G*), *rs17179108* (+3035 *G*/*A*), *rs9380142* (+3187 *T*/*C*) and *rs1610696* (+3196 *G*/*C*). The similarity and dissimilarity frequencies among the ZIKV non-transmitting mother–child pairs and the ZIKV-transmitting mother–child pairs are shown in Tables S2.1.–S2.3. (Supplementary Material S2) and represented below in Figure 1. There were no statistically significant associations of the genetic variants analyzed with ZIKV vertical transmission among the studied samples (see Supplementary Material S2).

### 3.2. Association of HLA-G and LILRB1/2 Polymorphisms in the ZIKV-Transmitting and Non-Transmitting Mothers and ZIKV-Infected and Non-Infected Children

To evaluate the possible associations between the *LILRB1*/*2* and *HLA-G* polymorphisms and the ZIKV-transmitting and non-transmitting mothers as well as the infected and non-infected children, a comparison of allele and genotype frequencies between these groups was carried out (Supplementary Material S3). The genotype distribution for all variants was in the HWE in the control groups. Regarding mothers, the *T*/*T* genotype related to the *LILRB1_rs1061684* polymorphism was denoted as a risk factor for the vertical transmission of ZIKV (*p* = 0.03, *Pc* = 0.06, OR = 12.4, CI = 1.91–391). The maternal *A*/*A* genotype and *A* allele referring to the *LILRB1_rs16985478* polymorphism also seem to influence the vertical transmission of ZIKV (*p* = 0.01, *Pc* = 0.02, OR = 19.2, CI = 1.71–643 and *p* = 0.03, *Pc* = 0.06, OR = 7.3, CI = 1.18–64.9, respectively). Additionally, the *G*/*A* genotype according to *LILRB2_rs386056* was denoted as a protective factor for vertical transmission. A significant difference was found using the overdominant genetic model (*p* = 0.02, *Pc* = 0.08, OR = 0.07, CI = 0.0–0.95) (Table 1).

In relation to the child analysis, the *T*/*T* genotype and *T* allele (*p* = 0.008, *Pc* = 0.016, OR = 16.4, CI = 1.8–471 and *p* = 0.01, *Pc* = 0.02, OR = 7.16, CI = 1.38–44.4, respectively) referring to the *LILRB1_rs1061684* polymorphism and the *A*/*A* genotype and *A* allele (*p* = 0.008, *Pc* = 0.016, OR = 16.4, CI = 1.8–471 and *p* = 0.01, *Pc* = 0.02, OR = 7.16, CI = 1.38–44.4, respectively) referring to the *LILRB1_rs16985478* polymorphism were denoted as risk factors for ZIKV infection due to vertical transmission. In contrast, the *G* allele (*p* = 0.02, *Pc* = 0.08, OR = 0.12, CI = 0.01–0.77) and *G*/*G* genotype (*p* = 0.01, *Pc* = 0.04, OR = 0.04, CI = 0.0–0.71) related to the *LILRB2_rs7247451* polymorphism were denoted as protective factors against ZIKV infection in children according to the dominant genetic model (Table 2).

Regarding the *HLA-G* gene analysis, it was observed that only the maternal *T* allele related to the *HLA-G_rs1710* polymorphism was associated as risk factor for the vertical transmission of ZIKV (*p* = 0.04; *Pc* = 0.52; OR = 4.30; CI = 1.005–20.9) (Table 1). Contrary, the *T*/*T* genotype *HLAG_rs9380142* was associated to protection against ZIKV congenital transmission (*p* = 0.04; *Pc* = 0.52; OR =0.14; CI =0.01–1.32), considering the Log-additive genetic model (Table 1). No other *HLA-G* variants showed to be significant in the mother’s allelic and genotypic comparisons. Similarly, no statistically significant differences were found for *HLA-G* gene polymorphisms between ZIKV-infected and non-infected children (Supplementary Material S3—Tables S3.3. and S3.4.).

## 4. Discussion

The identification of potential mutations in *HLA-G* and its high-affinity binding receptors, *LILRB1* and *LILRB2*, may improve the basic understanding of the impact of the *HLA-G* and *LILRB1*/*2* genes on in utero ZIKV transmission. Since HLA-G is expressed in maternal–fetal tissues and modulates immunity during pregnancy by binding to specific receptors with great affinity, it is possible that these molecules may play a role in virus transmission from an infected mother to her child [20]. HLA-G and its receptors (LILs) are the best studied molecules present in the placenta barrier and in viral infections that highly modulate the immune microenvironment. Flaviviruses, but also other viruses such HIV, take the same route to pass the maternal–fetal barrier and infect the developing child. It has been suggested that ZIKV and HIV induce tropism to cells of the placenta barrier, where HLA-G is highly present (i.e., Hoffbauer cells and trophoblasts) [21].

Polymorphisms in the cytoplasmatic immunoreceptor tyrosine-based inhibitory motif (ITIM) region of *LILRB1* may impact the appropriate inhibitory signaling or molecule stability [22]. SNPs in *LILRB2* may impair immunoglobulin domain binding (Ig-D) to HLA class I molecules or affect the splicing process [23]. Similarly, polymorphic sites in the *HLA-G exon 2–4* and *3′UTR* regions can affect the appropriate function (binding) of the alpha 1–3 domains or molecule stability and influence mRNA instability, splicing and expression of the HLA-G molecules [20]. The *3′UTR* is well known for presenting polymorphic sites that influence gene expression [24]. The well-studied *14bp INS*/*DEL* polymorphism, for example, shows lower mRNA production for most HLA-G membrane-bound and soluble isoforms in trophoblasts when the *3′UTR* sequence has the insertion of the 14bp segment [24]. Contrarily, the deletion of this 14bp segment leads to an increased expression of HLA-G molecules [24]. Additionally, the surrounding 14bp polymorphisms might contribute to the alternative splicing in *HLA-G*. Therefore, the presence of variants in the studied ligand–receptor genes can be implicated in the changes in molecule behavior and alter the immunological environment from an anti-inflammatory to a pro-inflammatory phenotype, thus favoring virus replication.

In our study, the similar/dissimilar investigation showed no evidence of a possible correlation indicating that genetic variants in the *HLA-G* and *LILRB1*/*2* genes could be implicated in intrauterine ZIKV infection, although the relatively small sample size of our study might be a possible bias. A study on HIV-1 transmission from mother to child with 194 pairs showed similar results to ours, in which there was no indication of the influence of mother–child *HLA-G* genotype similarity/dissimilarity on the risk of vertical transmission. That work analyzed polymorphisms in *codons 31*, *57*, *93*, *110* and *130* of *HLA-G exons 2* and *3* [25]. Another study also corroborates that variants in *HLA-G exons 2–3* and concordance between the mother and baby might not be associated with congenital HIV transmission [26]. Different from our results, it has already been reported that discordance between a mother and child for variants in *HLA-G exon 2* is associated with a reduced risk of HIV-1 transmission, while those pairs carrying the same genotype may have higher risks [27].

Alternatively, we also investigated the possible role of the *LILRB1*/*2* and *HLA-G* variants in congenital transmission between ZIKV-transmitting and non-transmitting mothers, independently from the ZIKV-infected and non-infected children groups. In these analyses, we observed an interesting association of the homozygous genotype variants of *LILRB1*_*rs1061684* and *LILRB1_rs16985478* as a susceptibility factor for congenital ZIKV transmission in the mother and children groups. These data suggest a strong association of these polymorphisms, with around 12–19 times higher chances of mother–child transmission or of the child being infected while in the mother’s womb. *LILRB1_rs1061684* is located very close to the non-synonym *LILRB1_16985478*—both are situated in the cytoplasmatic ITIM and are suggested to influence the appropriate inhibitory signaling or molecule stability. These polymorphisms, especially *LILRB1_16985478*, could impact ZIKV transmission in different manners given the broad pattern of LILRB1 expression in different immune cells. We highlight that even after BC, the *A*/*A* genotype of *LILRB1_16985478* was still considered a susceptibility factor for ZIKV transmissibility from mother to child.

Similarly, the polymorphic *HLA-G_ rs1710* allele was correlated as a potential susceptibility factor for ZIKV vertical transmission in the mothers, with four times higher chances of this event possibly occurring. In contrast, the wild-variant genotype of *HLAG_rs9380142* was associated with protection. However, significance was lost after BC for both polymorphisms analyzed. These variants are located in the *3′UTR* segment of the *HLA-G* gene. A study on HIV-1 suggested that variants in *exon 8* of the *HLA-G 3′UTR* with or without the participation of variants in *exon 2* codon 57 and *5′UTR* may play a role in virus vertical transmission [14]. In endometriosis, genetic variants in *HLA-G* and *LILRB1*/*2* are suggested to contribute to the development of the disease and its progression [23]. However, these studies did not investigate the *rs1710* polymorphism, although they do show the importance of the *3′UTR* polymorphisms.

Differently, *LILRB2*_*rs386056* and *LILRB2_rs7247451* were indicated to be protective genotypes in the mother and child groups, respectively. For *LILRB2_rs7247451*, the significance was maintained even after BC. As these polymorphisms are located in the Ig-D region, it is expected that these changes might weaken the binding levels between LILRB2 and HLA-G, consequently reducing the inhibitory signaling in myeloid lineage cells and controlling the immune response. Therefore, these *LILRB2* genotype variants could contribute to a protective outcome in mother–child ZIKV transmission. A study on LILRB2-HLA class I molecules verified that different binding levels between these molecules can result in different immune response outcomes and that a weak level of binding correlates with control of HIV-1 infection in a large cohort [28]. The lack of studies regarding *LILRB2* but also *LILRB1* polymorphisms in vertical transmission and viral infections makes it difficult to discuss the role of variants in this matter.

Mother–child HIV transmission is by far the most studied disease and topic of interest in investigations of virus vertical transmission, and HLA-G has also been frequently associated with this event, though this is possibly due to sample size bias. Therefore, the relationship of *HLA-G–LILRB1*/*2* polymorphisms has yet to be explored.

Given the contradictory data available in the literature on the role of *HLA-G* polymorphisms and the lack of studies on *LILRB1*/*2* genetic variants in viral infections and vertical transmission, new studies should be considered and encouraged to better comprehend the influence of these host factors on diseases.

## 5. Conclusions

The current study suggests that specific polymorphic sites, mainly *LILRB1* (*rs1061684* and *rs16985478*) and *HLA-G* (*rs1710*), could be associated with mother-to-child ZIKV transmission, while *LILRB2* (*rs386056* and *rs7247451*) could be a protective factor against ZIKV vertical transmission in a population from the south and southeast of Brazil. However, a larger cohort including people from different regions of Brazil needs to be considered to expand these findings and thus contribute to improving our knowledge of the factors involved in ZIKV vertical transmission.

## Figures and Tables

**Table 1:** Statistically significant allele and genotype distribution for *LILRB1*, *LILRB2* and *HLA-G* polymorphisms in mothers who vertically transmitted or not the ZIKV to their child.

MOTHERS					
ALLELES/GENOTYPES	M-ZIKV−	M-ZIKV+	OR (CI)	*p*-Value	*Pc*
	*N* = 6	*N* = 13 ^†^			
*LILRB1_rs1061684 C > T*					
	*n* = 6 (%)	*n* = 12 (%)			
C/T	5 (83)	3 (25)	12.4 (1.91–391)	0.03	0.06
T/T	1 (17)	9 (75)			
*LILRB1_rs16985478 G > A*					
	*n* = 6 (%)	*n* = 12 (%)			
G	5 (42)	2 (8)	7.3 (1.18–64.9)	0.03	0.06
A	7 (58)	22 (92)			
G/A	5 (83)	2 (17)	19.2 (1.71–643)	0.01	**0.02**
A/A	1 (17)	10 (83)			
*LILRB2_rs386056 A > G*					
	*n* = 6 (%)	*n* = 11 (%)			
A/A	0	1 (9)	0.07 (0.0–0.95) ^1^	0.02	0.08
G/A	5 (83)	3 (27)			
G/G	1 (17)	7 (64)			
*HLAG_rs1710 C > G*					
	*n* = 6 (%)	*n* = 13 (%)			
C	8 (67)	8 (31)	4.30 (1.005–20.9)	0.04	0.52
G	4 (33)	18 (69)			
*HLAG_rs9380142 T > C*					
	*n* = 6 (%)	*n* = 13 (%)			
T/T	1 (17)	7 (54)	0.14 (0.01–1.32) ^2^	0.04	0.52
T/C	4 (67)	6 (46)			
C/C	1 (17)	0			

M-ZIKV−: Mothers with no vertical transmission occurrence. M-ZIKV+: Mothers with vertical transmission occurrence. *Pc*: *p*-value adjusted by Bonferroni correction method. ^1^ Overdominant genetic model. ^2^ Log-additive genetic model. ^†^ The total number of genotyped subjects (*n*) is described for each polymorphism.

**Table 2:** Statistically significant allele and genotype distribution for *LILRB1* and *LILRB2* polymorphisms in ZIKV-infected and non-infected children due to vertical transmission.

CHILDREN					
ALLELES/GENOTYPES	C-ZIKV−	C-ZIKV+	OR (CI)	*p*-Value	*Pc*
	*N* = 6	*N* = 14 **^†^			
*LILRB1_rs1061684 C > T*					
	*n* = 6 (%)	*n* = 13 (%)			
T	6 (50)	23 (88)	7.16 (1.38–44.6)	0.01	**0.02**
C	6 (50)	3 (12)			
C/T	6 (100)	3 (23)	16.4 (1.8–471)	0.008	**0.016**
T/T	0	10 (77)			
*LILRB1_rs16985478 G > A*					
	*n* = 6 (%)	*n* = 13 (%)			
G	6 (50)	3 (12)	7.16 (1.38–44.6)	0.01	**0.02**
A	6 (50)	23 (88)			
G/A	6 (100)	3 (23)	16.4 (1.8–471)	0.008	**0.016**
A/A	0	10 (77)			
*LILRB2_rs7247451 G > C*					
	*n* = 6 (%)	*n* = 13 (%)			
G	7 (58)	24 (92)	0.12 (0.01–0.77)	0.02	0.08
C	5 (42)	2 (8)			
G/G	2 (33)	12 (92)	0.04 (0.0–0.71) ^1^	0.01	**0.04**
G/C	3 (50)	0			
C/C	1 (17)	1 (8)			

C-ZIKV−: Child tested negative for ZIKV. C-ZIKV+: Child tested positive for ZIKV. *Pc*: *p-*value adjusted by Bonferroni correction method. ^1^ Dominant genetic model. ** A pair of twins included. ^†^ The total number of genotyped subjects (*n*) is described for each polymorphism.

## Data Availability

Not applicable.

## Supplementary Materials

The following supporting information can be downloaded at: https://www.mdpi.com/article/10.3390/cimb44070191/s1. Supplementary Material S1—PCR and sequencing conditions. Table S1.1.: Polymerase-Chain Reaction Primer sequences for amplification of the *HLA-G* and *LILRB*s genes; Table S1.2. PCR thermocycle conditions for *HLA-G* and *LILRB*s genes; Table S1.3. Sanger Sequencing reaction cycle conditions for *HLA-G* and *LILRB*s genes. Supplementary Materials S2—*LILRB*s and *HLA-G* dissimilarity/similarity tables. Table S2.1.: *LILRB1* and *LILRB2* variant dissimilarities/similarities between Mother-child pairs and risk of vertical ZIKV transmission; Table S2.2. *HLA-G Exons 2-4* variant dissimilarities/similarities between Mother-child pairs and risk of vertical ZIKV transmission; Table S2.3. *HLA-G Exons 8* variant dissimilarities/similarities between Mother-child pairs and risk of vertical ZIKV transmission. Supplementary Materials S3—Association tables of *HLA-G* and *LILRB1*/*2* polymorphisms in the ZIKV-transmitting and non-transmitting mothers; ZIKV-infected and non-infected children. Table S3.1.: Allele and genotype distribution for *LILRB1*/*2* polymorphisms in mothers who vertically transmitted or not the ZIKV to their newborns; Table S3.2.: Allele and genotype distribution for *LILRB1*/*2* polymorphisms in infected and not infected children with ZIKV due to vertical transmission; Table S3.3.: Allele and genotype distribution for *HLA-G* polymorphisms in mothers who vertically transmitted or not the ZIKV to their newborns; Table S3.4.: Allele and genotype distribution for *HLA-G* polymorphisms in infected and not infected children with ZIKV due to vertical transmission; Table S3.5.: List of variants sites for *LILRB1*, *LILRB2* and *HLA-G*, SNP identification and function effects of each variant according to the NCBI-dbSNP.
