# Supplementary material for: HLA-G, LILRB1 and LILRB2 Variants in Zika Virus Transmission from Mother to Child in a Population from South and Southeast of Brazil
Source: Curr Issues Mol Biol. 2022 Jun 27;44(7):2783–93. doi: 10.3390/cimb44070191 (PMC9317030; doi:10.3390/cimb44070191)
Supplement: Supplementary file 1 [file cimb-44-00191-s001.zip › SM_S1_PCR and Sequencing Conditions.pdf]

Supplementary Material 1

PCR and Sequencing Conditions

Table S1.1. Polymerase-Chain Reaction Primer sequences for amplification of the *HLA-G* and *LILRB*s genes.

Gene	PRIMER ID	Primer Sequence (5'->3')	PRODUCT SIZE
HLA-G	HLA-GF1 **	F: 5' TCTCCTAACCTGTGTCGGGT 3'	1251pb
Exons 2-4	HLAGR1 ***	R: 5' ACCAGAGGGAGGGCGATATTC 3'	
HLA-G	HLA-GF3	F: 5' TGTGAAACAGCTGCCCTGTGT 3'	523pb
Exon 8	HLA-GR3 *	R: 5' CTGGTGGGACAAGGTTCTACTG 3'	
LILRB1	LILRB1F *	F: 5' CGGGAAAGGGATGTAATCGG 3'	613pb
	LILRB1R	R: 5' CCCTAAAAGTTCCCAGAGTCTCC 3'	
LILRB2	LILRB2F *	F: 5' CCCTATGTGTGGTCTTCACCC 3'	660pb
	LILRB2R	R: 5' TGGCCATCACTAATTGGATTCC 3'	

bp: base pair; *Primers with the lowest or no background, chosen after protocol optimization to sequence only one DNA strand for each gene region. ** Primer used to sequence the *HLA-G* regions of *Exons 2-3*. *** Primer used to sequence the *HLA-G* region of *Exon 4*.

Table S1.2. PCR thermocycle conditions for *HLA-G* and *LILRB*s genes.

Gene	Initial Denaturation	Denaturation/Tm/Extension	Final Extention
**N° Cycles**	**1x**	**35x**	**1x**
HLA-G *Exons 2-4*	96°C-15min	94°C – 30s/65°C–30s/72°C–30s	72ºC-10min
HLA-G *Exon 8*	95°C-5min	95°C – 45s/56°C–15s/72°C–30s	72ºC-7min
LILRB1	95°C-5min	95°C–30s/60°C–45s/72°C–30s	72ºC-10min
LILRB2	95°C-5min	95°C–30s/62°C – 30s/72ºC–30s	72ºC-2min

Table S1.3. Sanger Sequencing reaction cycle conditions for *HLA-G* and *LILRB*s genes.

Gene	PRIMER ID USED	Incubation	Denaturation/Tm/Extension	Product Size
**N° Cycles**		**1x**	**25x**	
HLA-G *Exons 2-3*	HLA-GF1	96°C-1min	96°C–10s/50°C–5s/72ºC–4min	775pb
HLA-G *Exon 4*	HLA-GR1	96°C-1min	96°C–10s/50°C–5s/72ºC–3min	497pb
HLA-G *Exon 8*	HLA-GR3	96°C-1min	96°C–10s/50°C–5s/72ºC–3min	523pb
LILRB1	LILRB1F	96°C-1min	96°C–10s/50°C–5s/72ºC–4min	613pb
LILRB2	LILRB2R	96°C-1min	96°C–10s/50°C–5s/72ºC–4min	660pb

Tm: Melting Temperature

Filename: SM_S1_PCR and Sequencing Conditions.docx
Directory: E:\5.12\ijms-D100
Template: C:\Users\MDPI\AppData\Roaming\Microsoft\Templates\Normal.dotm
Title:
Subject:
Author: Microsoft Office User
Keywords:
Comments:
Creation Date: 5/6/2022 4:29:00 PM
Change Number: 2
Last Saved On: 5/6/2022 4:29:00 PM
Last Saved By: Jelena Vakić
Total Editing Time: 6 Minutes
Last Printed On: 5/12/2022 5:45:00 PM
As of Last Complete Printing
Number of Pages: 1
Number of Words: 257 (approx.)
Number of Characters: 1,543 (approx.)
