# Supplementary material for: HLA-G, LILRB1 and LILRB2 Variants in Zika Virus Transmission from Mother to Child in a Population from South and Southeast of Brazil
Source: Curr Issues Mol Biol. 2022 Jun 27;44(7):2783–93. doi: 10.3390/cimb44070191 (PMC9317030; doi:10.3390/cimb44070191)
Supplement: Supplementary file 1 [file cimb-44-00191-s001.zip › SM_S2_LILRBs and HLAG similiar_dissimilar tables.pdf]

Supplementary Material 2

LILRBs **and** ***HLA-G*** **dissimilarity/similarity tables**

Table S2.1. *LILRB1* and *LILRB2* variant dissimilarities/similarities between Mother-child pairs and risk of vertical ZIKV transmission.

**Gene/SNP ID**		**ZIKV-Transmitting group N = 14** †	**ZIKV-Non-Transmitting group N = 6**	***p***	**OR (CI 95%)**
LILRB1					
		**n = 12 (%)**	**n = 6 (%)**		
rs1061684	Dissimilar	4 (30.7)	1 (16.7)	0.87	0.41 (0.067–6.15)
	Similar	8 (61.5)	5 (83.3)		
		**n = 12 (%)**	**n = 6 (%)**		
rs16985478	Dissimilar	4 (30.7)	1 (16.7)	0.87	0.41 (0.067–6.15)
	Similar	8 (61.5)	5 (83.3)		
LILRB2					
		**n = 11 (%)**	**n = 6 (%)**		
rs386056	Dissimilar	4 (30.8)	2 (33.3)	0.92	0.88 (0.55–10.27)
	Similar	7 (53.8)	4 (66.7)		
		**n = 11 (%)**	**n = 6 (%)**		
rs7247538	Dissimilar	5 (38.4)	4 (66.7)	0.75	2.27 (0.211–35.6)
	Similar	6 (41.6)	2 (33.3)		
		**n = 11 (%)**	**n = 6 (%)**		
rs7247451	Dissimilar	3 (23.0)	4 (66.7)	0.28	4.76 (0.42–81.3)
	Similar	8 (61.5)	2 (33.3)		
		**n = 11 (%)**	**n = 6 (%)**		
rs7247208	Dissimilar	6 (41.6)	3 (50.0)	0.28	4.68 (0.42–81.3)
	Similar	5 (38.4)	3 (50.0)		

† The total number of genotyped subjects (n) is described for each polymorphism

Table S2.2. *HLA-G Exons 2 - 4* variant dissimilarities/similarities between Mother-child pairs and risk of vertical ZIKV transmission.

Gene/SNP ID		ZIKV-Transmitting group N = 14 †	ZIKV-Non-Transmitting group N = 6 †	*p*	OR (CI 95%)
		HLA-G–Exon 2			
		**n = 9 (%)**	**n = 6 (%)**		
rs1630224	Dissimilar	7 (77.7)	5 (83.3)	0.84	0.71 (0.009–17.6)
	Similar	2 (22.3)	1 (16.7)		
		**n = 10 (%)**	**n = 6 (%)**		
rs1630185	Dissimilar	6 (60)	5 (83.3)	0.69	0.32 (0.005–4.88)
	Similar	4 (40)	1 (16.7)		
		HLA-G–Exon 3			
		**n = 9 (%)**	**n = 6 (%)**		
rs1130355	Dissimilar	5 (38.4)	5 (83.3)	0.58	0.27 (0.004–4.24)
	Similar	4 (30.8)	1 (16.7)		
		HLA-G–Exon 4			
		**n = 8 (%)**	**n = 5 (%)**		
rs1130356	Dissimilar	2 (55.6)	2 (40)	0.62	0.52 (0.25–10.81)
	Similar	6 (44.4)	3 (60)		
		**n = 8 (%)**	**n = 6 (%)**		
rs1632942	Dissimilar	5 (62.5)	3 (50.0)	0.68	1.6 (0.123–22.6)
	Similar	3 (37.5)	3 (50.0)		

† The total number of genotyped subjects (n) is described for each polymorphism

Table S2.3. *HLA-G Exons 8* variant dissimilarities/similarities between Mother-child pairs and risk of vertical ZIKV transmission.

Gene/SNP ID		ZIKV-Transmitting group N = 14 [†]	ZIKV-Non-Transmitting group N = 6	*p*	OR (CI 95%)
			HLA-G–Exon 8		
		**n = 13 (%)**	**n = 6 (%)**		
rs371194629	Dissimilar	8 (61.5)	3 (50.0)	0.67	1.56 (0.146–17.02)
	Similar	5 (38.5)	3 (50.0)		
		**n = 13 (%)**	**n = 6 (%)**		
rs1063320	Dissimilar	8 (61.5)	5 (83.3)	0.69	0.33 (0.05–4.56)
	Similar	5 (38.4)	1 (16.7)		
		**n = 13 (%)**	**n = 6 (%)**		
rs1707	Dissimilar	5 (38.4)	1 (16.7)	0.69	2.95 (0.28–88.2)
	Similar	8 (61.5)	5 (83.3)		
		**n = 13 (%)**	**n = 6 (%)**		
rs1710	Dissimilar	7 (54)	3 (50.0)	0.88	1.15 (0.109–12.3)
	Similar	6 (46)	3 (50.0)		
		**n = 13 (%)**	**n = 6 (%)**		
rs17179101	Dissimilar	2 (15.4)	2 (33.3)	0.74	0.38 (0.02–7.00)
	Similar	11 (84.6)	4 (66.7)		
		**n = 13 (%)**	**n = 6 (%)**		
rs17179108	Dissimilar	8 (61.5)	2 (33.3)	0.51	3.0 (0.29–45.3)
	Similar	5 (38.5)	4 (66.7)		
		**n = 13 (%)**	**n = 6 (%)**		
rs9380142	Dissimilar	7 (54)	3 (50.0)	0.88	1.15 (0.109–12.3)
	Similar	6 (46)	3 (50.0)		
		**n = 13 (%)**	**n = 6 (%)**		
rs1610696	Dissimilar	2 (15.4)	3 (50.0)	0.30	0.20 (0.01–2.6)
	Similar	11 (84.6)	3 (50.0)		

[†] The total number of genotyped subjects (n) is described for each polymorphism

Filename: SM_S2_LILRBs and HLAG similiar_dissimilar tables.docx
Directory: E:\5.12\ijms-D100
Template: C:\Users\MDPI\AppData\Roaming\Microsoft\Templates\Normal.dotm
Title:
Subject:
Author: Microsoft Office User
Keywords:
Comments:
Creation Date: 5/6/2022 4:29:00 PM
Change Number: 2
Last Saved On: 5/6/2022 4:29:00 PM
Last Saved By: Jelena Vakić
Total Editing Time: 3 Minutes
Last Printed On: 5/12/2022 5:50:00 PM
As of Last Complete Printing
Number of Pages: 3
Number of Words: 637 (approx.)
Number of Characters: 2,684 (approx.)
