# Supplementary material for: HLA-G, LILRB1 and LILRB2 Variants in Zika Virus Transmission from Mother to Child in a Population from South and Southeast of Brazil
Source: Curr Issues Mol Biol. 2022 Jun 27;44(7):2783–93. doi: 10.3390/cimb44070191 (PMC9317030; doi:10.3390/cimb44070191)
Supplement: Supplementary file 1 [file cimb-44-00191-s001.zip › SM_S3_TABLES HLAG_LILRB1_mother_child.pdf]

Supplementary Material 3

Association Tables of HLA-G and LILRB1/2 polymorphisms in the ZIKV-transmitting and non-transmitting mothers; ZIKV- infected and non-infected children.

Table S3.1. Allele and genotype distribution for *LILRB1/2* polymorphisms in mothers who vertically transmitted or not the ZIKV to their newborns.

	MOTHERS			
**ALLELES/GENOTYPES**	**M-ZIKV-**	**M-ZIKV+**	**OR (CI)**	***p*-Value**
	**N = 6**	**N = 13** [†]		
	LILRB1_rs1061684 C>T			
	n = 6 (%)	n = 12 (%)		
**T**	7 (58)	21 (88)	**4.74 (0.87–29.8)**	0.07 *
**C**	5 (42)	3 (12)		
**C/T**	5 (83)	3 (25)	**12.4 (1.91–391)**	**0.03 ***
**T/T**	1 (17)	9 (75)		
	LILRB1_rs16985478 G>A			
	n = 6 (%)	n = 12 (%)		
**G**	5 (42)	2 (8)	**7.3 (1.18–64.9)**	**0.03 ***
**A**	7 (58)	22 (92)		
**G/A**	5 (83)	2 (17)	**19.2 (1.71–643)**	**0.01 ***
**A/A**	1 (17)	10 (83)		
	LILRB2_rs386056 A>G			
	n = 6 (%)	n = 11 (%)		
**A**	5 (42)	5 (23)	2.4 (0.53–11.1)	0.44
**G**	7 (58)	17 (77)		
**A/A**	0	1 (9)		
**G/A**	5 (83)	3 (27)	**0.07 (0.0–0.95)** [3]	**0.02 ***
**G/G**	1 (17)	7 (64)		
	LILRB2_rs7247538 C>T			
	n = 6 (%)	n = 11 (%)		
**C**	8 (67)	9 (41)	2.7 (0.64–13.6)	0.17
**T**	4 (33)	13 (59)		
**C/C**	2 (33)	3 (27)		
**C/T**	4 (67)	3 (27)	4.76 (0.55–55) [3]	0.16
**T/T**	0	5 (45)		
	LILRB2_rs7247451 G>C			
	n = 6 (%)	n = 11 (%)		
**G**	7 (58)	17 (77)	0.41 (0.09–1.8)	0.44
**C**	5 (42)	5 (23)		
**G/G**	3 (50)	8 (73)	0.33 (0.04–2.8) [1]	0.55
**G/C**	1 (17)	1 (9)		

**C/C**	2 (33)	2 (18)		
LILRB2_rs7247208 T>C				
	n = 6 (%)	n = 11 (%)		
**T**	7 (58)	9 (41)	2.0 (0.48–8.4)	0.53
**C**	5 (42)	13 (59)		
**T/T**	2 (33)	3 (27)		
**T/C**	3 (50)	3 (27)	1.3 (0.11–12.7) [1]	0.8
**C/C**	1 (17)	5 (45)		

M-ZIKV-: Mothers with no vertical transmission occurrence; M-ZIKV+: Mothers with vertical transmission occurrence; * Mid-P exact test; [1] Dominant genetic Model; [2] Recessive genetic model; [3] Overdominant genetic model; [4] Log-additive genetic model. [†] The total number of genotyped subjects (n) is described for each polymorphism.

Table S3.2. Allele and genotype distribution for *LILRB1/2* polymorphisms in infected and not infected children with ZIKV due to vertical transmission.

	CHILDREN			
ALLELES/GENOTYPES	C-ZIKV-	C-ZIKV+	OR (CI)	*p*-Value
	N = 6	N = 14 ** †		
		LILRB1_rs1061684 C>T		
	n = 6 (%)	n = 13 (%)		
**T**	6 (50)	23 (88)	**7.16 (1.38–44.6)**	**0.01 ***
**C**	6 (50)	3 (12)		
**C/T**	6 (100)	3 (23)	**16.4 (1.8–471)**	**0.008 ***
**T/T**	0	10 (77)		
		LILRB1_rs16985478 G>A		
	n = 6 (%)	n = 13 (%)		
**G**	6 (50)	3 (12)	**7.16 (1.38–44.6)**	**0.01 ***
**A**	6 (50)	23 (88)		
**G/A**	6 (100)	3 (23)	**16.4 (1.8–471)**	**0.008 ***
**A/A**	0	10 (77)		
		LILRB2_rs386056 A>G		
	n = 6 (%)	n = 13 (%)		
**A**	5 (42)	4 (15)	3.7 (0.75–20.1)	0.1 *
**G**	7 (58)	22 (85)		
**A/A**	1 (17)	0		
**G/A**	3 (50)	4 (31)	5.4 (0.37–176)[4]	0.22
**G/G**	2 (33)	9 (69)		
		LILRB2_rs7247538 C>T		
	n = 6 (%)	n = 13 (%)		
**C**	10 (83)	13 (50)	4.8 (0.94–37.7)	0.06 *
**T**	2 (17)	13 (50)		
**C/C**	4 (67)	2 (15)		
**C/T**	2 (33)	9 (69)	8.76 (0.67–115)[4]	0.06
**T/T**	0	2 (15)		
		LILRB2_rs7247451 G>C		
	n = 6 (%)	n = 13 (%)		
**G**	7 (58)	24 (92)	**0.12 (0.01–0.77)**	**0.02 ***
**C**	5 (42)	2 (8)		
**G/G**	2 (33)	12 (92)		
**G/C**	3 (50)	0	**0.04 (0.0–0.71)[1]**	**0.01**
**C/C**	1 (17)	1 (8)		

		LILRB2_rs7247208 T>C		
	n = 6 (%)	n = 13 (%)		
**T**	8 (67)	13 (50)	2.0 (0.48–8.3)	0.36 *
**C**	4 (33)	13 (50)		
**T/T**	3 (50)	2 (15)		
**T/C**	2 (33)	9 (69)	4.3 (0.49–38.3)[3]	0.18
**C/C**	1 (17)	2 (15)		

C-ZIKV-: Child tested negative for ZIKV; C-ZIKV+: Child tested positive for ZIKV; * Mid-P exact test; [1] Dominant genetic Model; [2] Recessive genetic model; [3] Overdominant genetic model; [4] Log-additive genetic model. ** A pair of twins included. † The total number of genotyped subjects (n) is described for each polymorphism.

Table S3.3. Allele and genotype distribution for *HLA-G* polymorphisms in mothers who vertically transmitted or not the ZIKV to their newborns.

			MOTHERS						
**ALLELES/GENOTYPES**	**M-ZIKV-**	**M-ZIKV+**	**OR (CI)**	***p*-Value**	**ALLELES/GENOTYPES**	**M-ZIKV-**	**M-ZIKV+**	**OR (CI)**	***p*-Value**
	**N = 6**	**N = 13** †				**N = 6**	**N = 13** †		
	HLAG_rs1630224 G>A					***HLAG_rs1707 A>G***			
	**n =6 (%)**	**n =10 (%)**				**n =6 (%)**	**n =13 (%)**		
**G**	7 (58)	8 (40)	2.6 (0.57–12.0)	0.37	**A**	11 (92)	24 (92)	0.91 (0.06–29.3)	0.9 *
**A**	5 (42)	12 (60)			**G**	1 (8)	2 (8)		
**G/G**	2 (33)	2 (20)			**A/A**	5 (83)	11 (85)		
**G/A**	3 (50)	4 (40)	1.91 (0.15–24.5) [1]	0.60 *	**A/G**	1 (17)	2 (15)	0.93 (0.06–13.4)	0.96
**A/A**	1 (17)	4 (40)			**G/G**	0	0		
	HLAG_rs1630185 G>A					***HLAG_rs1710 C>G***			
	**n =6 (%)**	**n =10 (%)**				**n =6 (%)**	**n =13 (%)**		
**G**	7 (58)	10 (50)	1.4 (0.33–5.9)	0.9	**C**	8 (67)	8 (31)	**4.30 (1.005–20.9)**	**0.04 ***
**A**	5 (42)	10 (50)			**G**	4 (33)	18 (69)		
**G/G**	2 (33)	4 (40)			**C/C**	3 (50)	1 (8)		
**G/A**	3 (50)	2 (20)	3.63 (0.36–44.7) [3]	0.27 *	**C/G**	2 (33)	6 (46)	3.99 (0.4–117) [2]	0.27 *
**A/A**	1 (17)	4 (40)			**G/G**	1 (17)	6 (46)		
	HLAG_rs1130355 G>A					***HLAG_rs17179101 T>G***			
	**n =6 (%)**	**n =10 (%)**				**n =6 (%)**	**n =12 (%)**		
**G**	7 (58)	9 (45)	1.7 (0.40–7.2)	0.7	**T**	3 (25)	4 (15)	1.80 (0.28–10.5)	0.5 *
**A**	5 (42)	11 (55)			**G**	9 (75)	22 (85)		
**G/G**	2 (33)	4 (40)			**T/T**	1 (17)	2 (15)		
**G/A**	3 (50)	1 (10)	7.6 (0.59–260.1) [3]	0.12 *	**T/G**	1 (17)	0	1.31 (0.12–11.2) [1]	0.7 *
**A/A**	1 (17)	5 (50)			**G/G**	4 (67)	11 (85)		
	HLAG_rs1130356 C>T					***HLAG_rs17179108 G>A***			
	**n =6 (%)**	**n =11 (%)**				**n =6 (%)**	**n =13 (%)**		
**C**	9 (90)	16 (73)	3.26 (0.39–86.2)	0.3 *	**G**	9 (75)	16 (62)	1.8 (0.40–10.2)	0.45 *
**T**	1 (10)	6 (27)			**A**	3 (25)	10 (38)		
**C/C**	4 (80)	7 (64)		0.39 *	**G/G**	4 (67)	5 (38)	4.9 (0.42–57.3) [3]	0.17 *

**C/T**	1 (20)	2 (18)	2.15 (0.30–15.19) [4]	
**T/T**	0	2 (18)		
HLAG_rs1632942 T>C				
	**n =6 (%)**	**n =11 (%)**		
**T**	9 (75)	12 (55)	2.43 (0.52–13.8)	0.2 *
**C**	3 (25)	10 (45)		
**T/T**	4 (67)	6 (55)		
**T/C**	1 (17)	0	3.64 (0.29–45.8)	0.29 *
**C/C**	1 (17)	5 (45)		
HLAG_rs371194629 INS/DEL				
	**n =6 (%)**	**n =13 (%)**		
**INS**	5 (42)	14 (54)	1.63 (0.40–6.5)	0.72
**DEL**	7 (58)	12 (46)		
**INS/INS**	1 (17)	5 (38)		
**INS/DEL**	3 (50)	4 (31)	1.42 (0.40–5.0) [4]	0.58 *
**DEL/DEL**	2 (33)	4 (31)		
HLAG_rs1063320 G>C				
	**n =6 (%)**	**n =13 (%)**		
**G**	7 (58)	12 (46)	1.63 (0.40–6.5)	0.72
**C**	5 (42)	14 (54)		
**G/G**	2 (33)	1 (8)		
**G/C**	3 (50)	6 (46)	6.53 (0.42–222) [4]	0.18 *
**C/C**	1 (17)	6 (46)		

**G/A**	1 (17)	6 (46)		
**A/A**	1 (17)	2 (15)		
HLAG_rs9380142 T>C				
	**n =6 (%)**	**n =13 (%)**		
**T**	6 (50)	20 (77)	0.3 (0.07–1.2)	0.19
**C**	6 (50)	6 (23)		
**T/T**	1 (17)	7 (54)		
**T/C**	4 (67)	6 (46)	0.14 (0.01–1.32) [4]	0.04 *
**C/C**	1 (17)	0		
HLAG_rs1610696 G>C				
	**n =6 (%)**	**n =13 (%)**		
**G**	10 (83)	20 (77)	1.48 (0.25–12.3)	0.7 *
**C**	2 (17)	6 (23)		
**G/G**	4 (67)	8 (62)		
**G/C**	2 (33)	4 (31)	1.19 (0.17–7.6) [3]	0.84
**C/C**	0	1 (8)		

M-ZIKV-: Mothers with no vertical transmission occurrence; M-ZIKV+: Mothers with vertical transmission occurrence; * Mid-P exact test; [1] Dominant genetic Model; [2] Recessive genetic model; [3] Overdominant genetic model; [4] Log-additive genetic model. † The total number of genotyped subjects (n) is described for each polymorphism.

Table S3.4. Allele and genotype distribution for *HLA-G* polymorphisms in infected and not infected children with ZIKV due to vertical transmission.

	CHILDREN								
ALLELES/GENOTYPES	C-ZIKV-	C-ZIKV+	OR (CI)	*p*-Value	ALLELES/GENOTYPES	C-ZIKV-	C-ZIKV+	OR (CI)	*p*-Value
	N = 6	N = 14 ** ǂ				N = 6	N = 14 ** ǂ		
	HLAG_rs1630224 G>A					*HLAG_rs1707 A>G*			
	**n =6 (%)**	**n =11 (%)**				**n =6 (%)**	**n =14 (%)**		
**G**	6 (50)	14 (64)	0.57 (0.13–2.37)	0.68	**A**	12 (100)	25 (89)	2.24 (0.17–80.9)	0.59 *
**A**	6 (50)	8 (36)			**G**	0	3 (11)		
**G/G**	2 (33)	6 (55)			**A/A**	6 (100)	11 (79)		
**G/A**	2 (33)	2 (18)	0.42 (0.05–3.31) [1]	0.4	**A/G**	0	3 (21)	----	0.12
**A/A**	2 (33)	3 (27)			**G/G**	0	0		
	HLAG_rs1630185 G>A					*HLAG_rs1710 C>G*			
	**n =6 (%)**	**n =12 (%)**				**n =6 (%)**	**n =14 (%)**		
**G**	6 (50)	16 (67)	0.5 (0.12–2.05)	0.54	**C**	7 (58)	12 (43)	1.86 (0.47–7.34)	0.58
**A**	6 (50)	8 (33)			**G**	5 (42)	16 (57)		
**G/G**	2 (33)	7 (58)			**C/C**	3 (50)	3 (21)		
**G/A**	2 (33)	2 (17)	0.36 (0.05–2.77) [1]	0.31	**C/G**	1 (17)	6 (43)	1.10 (0.13–11.3) [2]	0.94 *
**A/A**	2 (33)	3 (25)			**G/G**	2 (33)	5 (36)		
	HLAG_rs1130355 G>A					*HLAG_rs17179101 T>G*			
	**n =6 (%)**	**n =11 (%)**				**n =6 (%)**	**n =14 (%)**		
**G**	6 (50)	13 (59)	0.69 (0.16–2.85)	0.88	**T**	1 (8)	6 (21)	2.93 (0.36–75.4)	0.37 *
**A**	6 (50)	9 (41)			**G**	11 (92)	22 (79)		
**G/G**	2 (33)	6 (55)			**T/T**	0	3 (21)		
**G/A**	2 (33)	1 (9)	0.20 (0.01–2.88) [3]	0.22	**T/G**	1 (17)	0	1.36 (0.11–16.5) [1]	0.8 *
**A/A**	2 (33)	4 (36)			**G/G**	5 (83)	11 (79)		
	HLAG_rs1130356 C>T					*HLAG_rs17179108 G>A*			
	**n =6 (%)**	**n =11 (%)**				**n =6 (%)**	**n =14 (%)**		
**C**	9 (90)	20 (91)	0.90 (0.06–29.36)	0.9 *	**G**	11 (92)	18 (64)	5.89 (0.81–145.1)	0.08 *
**T**	1 (10)	2 (9)			**A**	1 (8)	10 (36)		

**C/C**	4 (80)	10 (91)		
**C/T**	1 (20)	0	0.40 (0.02– 8.07) [1]	0.55
**T/T**	0	1 (9)		
HLAG_rs1632942 T>C				
	**n =6 (%)**	**n =10 (%)**		
**T**	6 (50)	12 (60)	0.66 (0.15–2.82)	0.85
**C**	6 (50)	8 (40)		
**T/T**	2 (33)	6 (60)		
**T/C**	2 (33)	0	0.33 (0.04–2.77) [1]	0.3
**C/C**	2 (33)	4 (40)		
HLAG_rs371194629 INS/DEL				
	**n =6 (%)**	**n =14 (%)**		
**INS**	6 (50)	15 (54)	0.86 (0.22–3.35)	0.89
**DEL**	6 (50)	13 (46)		
**INS/INS**	2 (33)	4 (29)		
**INS/DEL**	2 (33)	7 (50)	0.51 (0.05–3.94) [3]	0.5
**DEL/DEL**	2 (33)	3 (21)		
HLAG_rs1063320 G>C				
	**n =6 (%)**	**n =14 (%)**		
**G**	5 (42)	9 (32)	1.5 (0.37–6.08)	0.82
**C**	7 (58)	19 (68)		
**G/G**	2 (33)	1 (7)		
**G/C**	1 (17)	7 (50)	1.13 (0.16–10.2) [2]	0.79
**C/C**	3 (50)	6 (43)		

**G/G**	5 (83)	7 (50)		
**G/A**	1 (17)	4 (29)	4.19 (0.53–32.9) [4]	0.09 *
**A/A**	0	3 (21)		
HLAG_rs9380142 T>C				
	**n =6 (%)**	**n =14 (%)**		
**T**	7 (58)	21 (75)	0.46 (0.11–1.95)	0.49
**C**	5 (42)	7 (25)		
**T/T**	2 (33)	7 (50)		
**T/C**	3 (50)	7 (50)	0.37 (0.06–2.10) [4]	0.24
**C/C**	1 (17)	0		
HLAG_rs1610696 G>C				
	**n =6 (%)**	**n =14 (%)**		
**G**	9 (75)	22 (79)	0.82 (0.16–4.80)	0.8 *
**C**	3 (25)	6 (21)		
**G/G**	4 (67)	8 (57)		
**G/C**	1 (17)	6 (43)	0.77 (0.11–4.64) [1]	0.79 *
**C/C**	1 (17)	0		

C-ZIKV-: Child tested negative for ZIKV; C-ZIKV+: Child tested positive for ZIKV ; * Mid-P exact test; [1] Dominant genetic Model; [2] Recessive genetic model; [3] Overdominant genetic model; [4] Log-additive genetic model. ** A pair of twins included. [†] The total number of genotyped subjects (n) is described for each polymorphism.

Table S3.5. List of variants sites for *LILRB1*, *LILRB2* and *HLA-G* , SNP identification and function effects of each variant according to the NCBI-dbSNP

SNP Nº	Gene_SNPID	Annotation *	SNP Nº	Gene_SNPID	Annotation *
**SNP1**	*LILRB1*_rs1061684	**synonymous**	**SNP11**	*HLAG*_rs1632942	**Intronic**
**SNP2**	*LILRB1*_rs16985478	**Non-synonymous**	**SNP12**	*HLAG_UTR*_rs371194629	**3 Prime UTR Variant**
**SNP3**	*LILRB2*_rs386056	**Non-synonymous**	**SNP13**	*HLAG_UTR*_rs1063320	**3 Prime UTR Variant**
**SNP4**	*LILRB2*_rs7247538	**Non-synonymous**	**SNP14**	*HLAG_UTR*_rs1707	**3 Prime UTR Variant**
**SNP5**	*LILRB2*_rs7247451	**Non-synonymous**	**SNP15**	*HLAG_UTR*_rs1710	**3 Prime UTR Variant**
**SNP6**	*LILRB2*_rs7247208	**synonymous**	**SNP16**	*HLAG_UTR*_rs17179101	**3 Prime UTR Variant**
**SNP7**	*HLAG*_rs1630224	**synonymous**	**SNP17**	*HLAG_UTR*_rs17179108	**3 Prime UTR Variant**
**SNP8**	*HLAG*_rs1630185	**synonymous**	**SNP18**	*HLAG_UTR*_rs9380142	**3 Prime UTR Variant**
**SNP9**	*HLAG*_rs1130355	**synonymous**	**SNP19**	*HLAG_UTR*_rs1610696	**3 Prime UTR Variant**

**SNP10**	*HLAG*_rs1130356	**synonymous**	

* Information collected from the dbSNP Short Genetic Variations of the NCBI (National Center for Biotechnology Information) database.

Filename: SM_S3_TABLES HLAG_LILRB1_mother_child.docx
Directory: E:\5.12\ijms-D100
Template: C:\Users\MDPI\AppData\Roaming\Microsoft\Templates\Normal.dotm
Title:
Subject:
Author: Microsoft Office User
Keywords:
Comments:
Creation Date: 5/6/2022 4:29:00 PM
Change Number: 2
Last Saved On: 5/6/2022 4:29:00 PM
Last Saved By: Jelena Vakić
Total Editing Time: 11 Minutes
Last Printed On: 5/12/2022 6:01:00 PM
As of Last Complete Printing
Number of Pages: 10
Number of Words: 2,274 (approx.)
Number of Characters: 8,509 (approx.)
